# Molecular epidemiology of HIV-1 infection among men who have sex with men in Taiwan from 2013 to 2015

**DOI:** 10.1371/journal.pone.0202622

**Published:** 2018-12-06

**Authors:** Wei-You Li, Marcelo Chen, Szu-Wei Huang, I-An Jen, Sheng-Fan Wang, Jyh-Yuan Yang, Yen-Hsu Chen, Yi-Ming Arthur Chen

**Affiliations:** 1 Center for Infectious Disease and Cancer Research, Kaohsiung Medical University, Kaohsiung, Taiwan; 2 Graduate Institute of Medicine, Kaohsiung Medical University, Kaohsiung, Taiwan; 3 Department of Urology, Mackay Memorial Hospital, Taipei, Taiwan; 4 Department of Cosmetic Applications and Management, Mackay Junior College of Medicine, Nursing and Management, Taipei, Taiwan; 5 Department and Institute of Public Health, National Yang-Ming University, Taipei, Taiwan; 6 Department of Medical Laboratory Science and Biotechnology, Kaohsiung Medical University, Kaohsiung, Taiwan; 7 Research and Diagnostic Center, Taipei, Centers for Disease Control, Department of Health, Taipei, Taiwan; 8 Division of Infectious Diseases, Department of Internal Medicine, Kaohsiung Medical University Hospital, Kaohsiung Medical University, Kaohsiung, Taiwan; 9 Institute of Biomedical Sciences, National Sun Yat-sen University, Kaohsiung, Taiwan; Fudan University, CHINA

## Abstract

Men who have sex with men (MSM) is the major risk population of HIV-1 infection in Taiwan, and its surveillance has become critical in HIV-1 prevention. We recruited MSM subjects from 17 high-risk venues and 4 community centers in northern and southern Taiwan for anonymous HIV-1 screening during 2013–2015. Blood samples were obtained for genotyping and phylogenetic analysis, and a questionnaire survey covering demographic variables and social behavior was conducted. In total, 4,675 subjects were enrolled, yielding a HIV-1 prevalence rate of 4.3% (201/4675). Eight risk factors including subjects who did not always use condoms (OR = 1.509, p = 0.0123), those who used oil-based lubricants (OR = 1.413, p = 0.0409), and those who used recreational drugs (OR = 2.182, p = < .0001) had a higher risk of HIV-1 infection. The annual prevalence and incidence of HIV-1 showed a downward trend from 2013 to 2015 (6.56%, 5.97 per 100 person-years in 2013; 4.53%, 3.97 per 100 person-years in 2014; 1.84%, 2.08 per 100 person-years in 2015). Factors such as always using condoms, water-based lubricant use, correct knowledge of lubricating substitutes, and recreational drug use were significantly associated with the trend of incidence. Phylogenetic tree analysis showed that the cross-regional and international interaction of the local MSM population may have facilitated transmission of HIV. This survey of high-risk venues showed decreased prevalence and incidence of HIV-1 infection in Taiwan from 2013 to 2015, and this may be related to changes in behavioral patterns. Moreover, cross-regional interaction and recreational drug use need to be considered in future surveillance.

## Introduction

The incidence of HIV-1 infection among men who have sex with men (MSM) has been increasing across the world, even in high-income countries [1–3]. After the explosive outbreak of HIV-1 among injection drug users (IDUs) occurred in Taiwan in 2004 and was controlled by the government, MSM have re-emerged as the major risk population in Taiwan since 2008 [4]. As of 2017, there were over 35,935 cases of HIV-1 infection in Taiwan, of which 62.31% were MSM, followed by IDUs (19.54%) and heterosexuals (16.6%) [5].

Our previous study showed that most of MSM infected with HIV-1 subtype B, and phylogenetic tree analysis showed that they were clustered with former isolated local strains in 2012 in Taiwan [6]. Along with other well-known factors associated with HIV-1 infection such as unprotected anal sex, sexual frequency, number of sexual partners, and history of sexually transmitted infection (STI), we also found that the misuse of oil-based solutions as lubricants was a new risk factor related to HIV-1 infection among MSM [6–8]. Based on these findings, educating at-risk populations how to recognize oil-based lubricants has become a new direction of intervention for HIV-1 prevention in Taiwan.

The increasing use of recreational drugs among MSM in Taiwan has also become an urgent issue to be addressed [9]. Similar trends have also been reported in other countries [10, 11]. The association of HIV-1 infection and recreational drugs has been well proved [12], and it may facilitate or become an excuse for unprotected sex [13, 14]. Moreover, the use of multiple recreational drugs among MSM and its association with HIV-1 infection has been reported [3, 15].

While surveillance of the MSM population has become critical in HIV-1 prevention in Taiwan, the challenge is that this critical population is also a hard-to-reach population due to its own complicated inherent issues such as stigmatization, and is thus considered as deviant [16, 17]. In order to overcome this issue, we used a time-location sampling method to investigate this hard-to-reach population in several commercial and non-commercial social venues. Following a cross-sectional study of HIV-1 epidemiology in Taiwan in 2012 [6], we conducted this three-year surveillance study to determine the trends of HIV-1 prevalence rate and incidence rate in 2013 to 2015, and to evaluate the risk factors among MSM infected with HIV-1 in Taiwan.

## Materials and methods

From 2013 to 2015, we cooperated with six gay bars, six gay saunas, five party events, and four community centers in northern (Taipei City and New Taipei City) and southern (Kaohsiung City) Taiwan and conducted time-location sampling. HIV-1 infected patients in these regions account for approximately half of the HIV-1 infected population in Taiwan [5]. Anonymous HIV-1 screening (including laboratory diagnosis and rapid test kit), blood samples and questionnaires were obtained from subjects at these venues. This study was approved by the institutional review board of Kaohsiung Medical University Chung-Ho Memorial Hospital (KMUHIRB-20130074, KMUHIRB-20140074) and Mackay Memorial Hospital (13MMHIS039).

### Determination of HIV-1 genotypes

Participants’ blood samples were drawn, and peripheral blood monocytes (PBMC) were extracted from those with positive HIV-1 screening results for virus genotyping. The proviral *gag*-gene regions were amplified by PCR. Nested multiplex PCR was performed on the basis of rTaq DNA Polymerase (TOYOBO CO., LTD, Japan), and the primers were designed by the subtype-specific segment in the *gag* region based on a previous established HIV-1 genotyping method [18]. Different HIV-1 subtypes produce product segments of different sizes, and HIV-1 subtypes can be determined by the segment size. Once multiplex PCR showed two or more HIV-1 subtypes suggesting dual infection, serial PCR using a single pair of subtype-specific primers was used for confirmation of dual infection.

### Phylogenetic tree analysis

Phylogenetic transmission clusters among MSM infected with HIV-1 was determined using phylogenetic tree analysis by maximum likelihood (ML) methods. The reagents and procedures of the nested multiplex PCR were described in a previous study [6], and the specific primers was designed with the HIV-1 *env* region (C2-V5). HIV-1 *env* sequences obtained from Sanger sequencing of PCR products using ABI 3730 DNA Analyzer (Applied Biosystems, US). The obtained sequence was aligned with the BioEdit v7.0.9.0 [19] software and the distance was calculated with an appropriate statistical model with the MEGA 6.0 software [20]. ML methods was used to conduct a consensus phylogenetic tree after bootstrap value analysis with 1,000 replicates. Genetic similarities and transmission networks were determined among MSM infected with HIV-1 in different regions of Taiwan. The reference viral subtype strains used for comparison were taken from the Los Alamos HIV sequence database (http://hiv-web.lanl.gov/).

### Nucleotide sequence accession numbers

The HIV-1 *env* sequences from 49 MSM patients used in phylogenetic analysis were deposited in GenBank (MH188084—MH188132).

### LAg-Avidity EIA test and incidence rate calculation

LAg-Avidity EIA test was performed according to the detailed test procedures and conditions in the product manual. The patients’ plasma samples were separated from the whole blood samples for the LAg-Avidity EIA test (Sedia Biosciences Corporation Portland, OR, USA). The infection time course of the patients was verified for calculation of the annual HIV-1 incidence rate.

### Statistical analysis

The behavioral risk factors related to HIV-1 infection were analyzed. Univariate and multivariate logistic regression analyses were used to estimate the effect of each variable. The Cochran-Armitage Trend Test was also used to analyze the trends of each risk factor through time. The data analysis for this paper was generated using SAS software, Version 9.4 of the SAS System for Windows. (Cary, NC, USA).

## Results

In total of the 4,675 participants, 201 were HIV-1 positive (201/4,675, 4.3%). The prevalence was 4.3% and the incidence rate was 4.04 per 100 person-years. Yearly statistics were as follows: in the 1,632 participants recruited in 2013, the prevalence was 6.56% (107/1,632) and the incidence rate was 5.97 per 100 person-years; in the 1,413 participants recruited in 2014, the prevalence was 4.53% (64/1,413) and the incidence rate was 3.97 per 100 person-years; in the 1,630 participants recruited in 2015, the prevalence was 1.84% (30/1,630) and the incidence rate was 2.08 per 100 person-years (Table 1). There was a trend towards dramatically lower prevalence and incidence rates with time (Table 2). From the viral subtype analysis results, the new HIV-1 positive infections were still mainly subtype B (158/201, 78.61%). One interesting note is that almost 10% of the infected subjects in 2013 were CRF01_AE, but this dropped to 1.56% and 0% in 2014 and 2015, respectively (Table 2).

**Table 1:** Demographic data of patrons from different gay venues participated in this study.

	HIV-1 (+)	HIV-1 (-)	Total	
Variable	N = 201	N = 4474	N = 4675	P
	n (%)	n (%)	n (%)	
**Years**				
**2013**	107 (53.2)	1525 (34.1)	1632 (34.9)	<0.0001^†^
**2014**	64 (31.8)	1349 (30.2)	1413 (30.2)	
**2015**	30 (14.9)	1600 (35.8)	1630 (34.9)	
**Area**				
**North of Taiwan**	159 (79.1)	3229 (72.2)	3388 (72.5)	0.0355^†^
**South of Taiwan**	42 (20.9)	1245 (27.8)	1287 (27.5)	
**Age**				
**18–29**	105 (52.2)	2251 (50.3)	2356 (50.4)	0.9932^†^
**30–39**	58 (28.9)	1314 (29.4)	1372 (29.3)	
**40–49**	17 (8.5)	416 (9.3)	433 (9.3)	
**≧50**	6 (3)	150 (3.4)	156 (3.3)	
**NA**	15 (7.5)	343 (7.7)	358 (7.7)	
**Occupation**				
**Student**	36 (17.9)	768 (17.2)	804 (17.2)	0.7199*
**Government employees**	22 (11.0)	404 (9)	426(9.1)	
**Office worker**	112 (55.7)	2504 (56)	2619 (56)	
**Specialist**	7 (3.5)	267 (6)	274 (5.9)	
**Unemployed/Other**	21 (10.5)	467 (10.4)	488 (10.4)	
**NA**	3 (1.5)	64 (1.4)	67 (1.4)	
**Education**				
**≤Junior high school**	8 (4)	84 (1.9)	92 (2)	0.0012^†^
**Senior high school**	48 (23.9)	740 (16.5)	788 (16.9)	
**College**	128 (63.7)	2976 (66.5)	3104 (66.4)	
**≧Graduate**	14 (7)	613 (13.7)	627 (13.4)	
**NA**	3 (1.5)	61 (1.4)	64 (1.4)	
**Marital status**				
**Single**	174 (86.6)	4166 (93.1)	4340 (92.8)	0.0007^†^
**Married**	5 (2.5)	107 (2.4)	112 (2.4)	
**Divorced/Separated/Widowed**	6 (3)	81 (1.8)	87 (1.9)	
**NA**	16 (8)	120 (2.7)	136 (2.9)	
**Sexual orientation**				
**Homosexual**	187 (93)	2655 (81.7)	3842 (82.2)	<0.0001^†^
**Bisexual**	9 (4.5)	664 (14.8)	673 (14.4)	
**NA**	5 (2.5)	155 (3.5)	160 (3.4)	
**Venue**				
**Gay saunas**	43 (21.4)	1338 (29.9)	1381 (29.5)	<0.0001^†^
**Gay night-clubs**	122 (60.7)	1957 (43.7)	2079 (44.5)	
**Party event**	26 (12.9)	268 (6)	294 (6.3)	
**Community centers**	10 (5)	911 (20.4)	921 (19.7)	

NA, Not available.

*, Chi-square test.

^†^, Fisher exact test.

**Table 2:** Prevalence, incidence rates and subtypes of HIV-1 infection among MSM in 2013–2015 in Taiwan.

	2013	2014	2015	Total
**No. of participants screened**	1632	1413	1630	4675
**No. of HIV-1 positive (%)**	107 (6.56)	64 (4.53)	30 (1.84)	201 (4.3)
No. of recent seroconverters^*^ **(% in total available sample)**	33/94 (35.11)	20/56 (35.71)	12/26 (46.15)	65/176 (36.93)
**Incidence rate (per 100 person-years)** ^**†**^	5.97	3.97	2.08	4.04
**Subtype**				
**B n (%)**	85 (79.44)	51 (79.69)	22 (73.33)	158 (78.61)
**CRF01_AE n (%)**	10 (9.35)	1 (1.56)	0 (0)	11 (5.47)
**B + CRF01_AE n (%)**	0 (0)	0 (0)	2 (6.67)	2 (1)
**NA and indeterminate**	12 (11.21)	12 (18.75)	6 (20)	30 (14.93)

*, Determined by Lag-Avidity EIA.

^†^, The index R used in incidence formula was adjusted by the percentage of recent seroconverters.

NA = Not available, including the participants of rapid test kit, and indeterminate genotyping result

**Fig 1:**
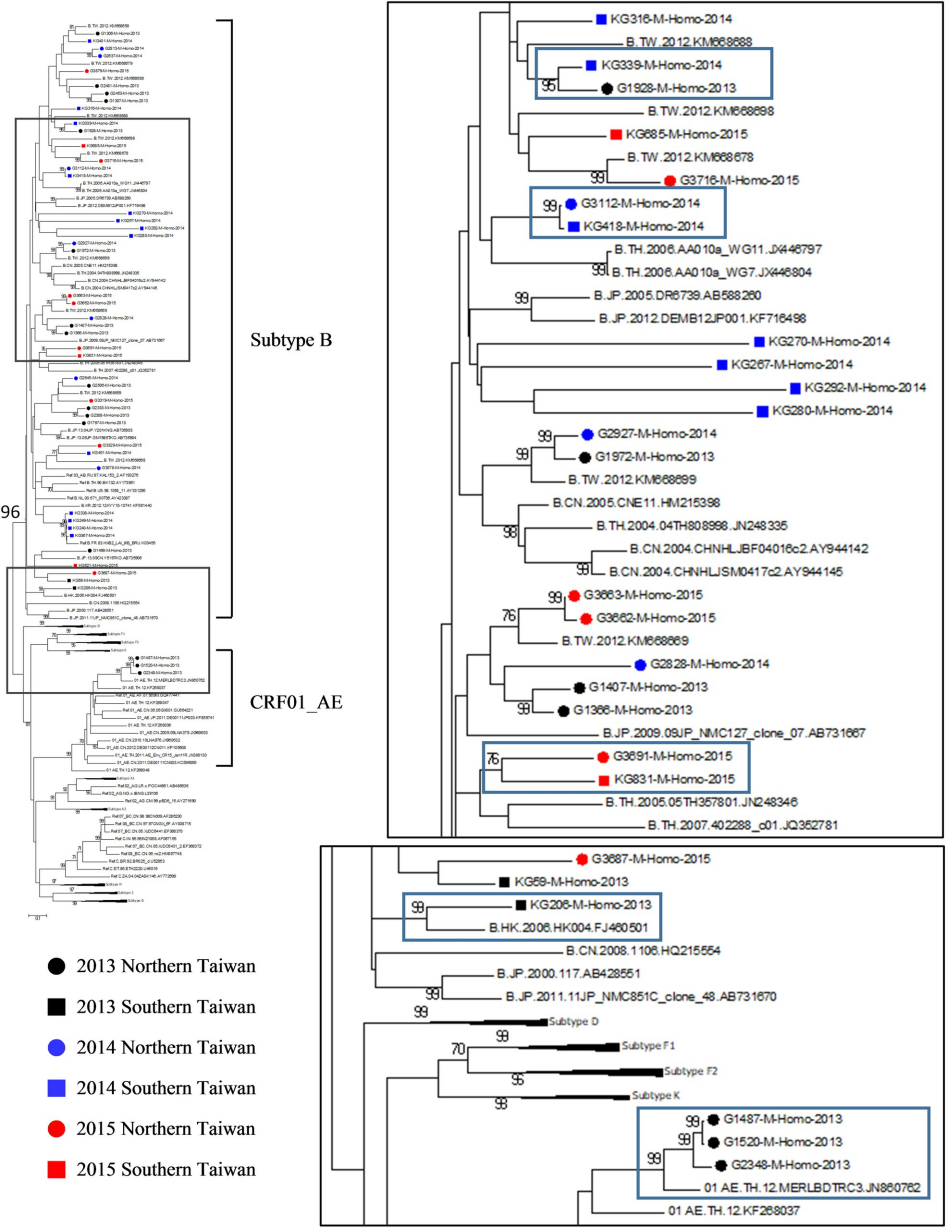
Phylogenetic analysis of HIV-1 strains identified from MSM in Taiwan during 2013–2015. Maximum-Likelihood (ML) tree analysis using *env* sequences based on the reference sequences from Los Alamos HIV sequence database. The evolutionary distances were computed using the GTR + G+I method.

Phylogenetic tree analysis showed that the transmission relationship between the MSM populations of southern and northern Taiwan located in the same cluster (KG339-2014 and G1928-2013, bootstrap value = 95, KG418-2014 and G3112-2014, bootstrap value = 99; KG831-2015 and G3691-2015, bootstrap value = 76) (Fig 1). In addition, patient KG206-2013 had a mutually clustered phenomenon with the 2006 Hong Kong reference strain (bootstrap value = 99). Three CRF01_AE subjects (G1487, G1520, G2348) in 2013 were clustered with 2012 Thailand reference strain (bootstrap value = 99). This suggested the possibility of international virus transmission in the present MSM infected with HIV-1 population.

Univariate and multivariate logistic regression analyses (Table 3) were performed to investigate the association between possible risk factors and HIV-1 infection. There was no significant association between the risk of HIV-1 infection and geographic location. Subjects with homosexual orientation had a higher probability of HIV-1 infection than bisexual subjects (OR = 2.617, p = 0.0043). Participants screened at gay saunas, gay bars, and party events had significantly higher risk of HIV-1 infection than those screened at community centers (OR = 2.742, p = 0.0053; OR = 5.116, p<0.0001; OR = 6.406, p<0.0001, respectively). Subjects with exclusively receptive roles and versatile roles during anal intercourse had higher risk of HIV-1 infection compared to those with exclusively insertive roles (OR = 3.606, p<0.0001; OR = 2.681, p<0.0001, respectively). Subjects with more than one sexual partner were more likely to be infected with HIV-1 than those with only one sexual partner (OR = 1.507, p = 0.0399). Subjects who did not always use condoms during sexual intercourse were more likely to be infected with HIV-1 than those who always used condoms (OR = 1.509, p = 0.0123). Those who used oil-based lubricants during sex had higher HIV-1 infection risk than those who used water-based lubricants (OR = 1.413, p = 0.0409). Moreover, participants with a history of other sexually transmitted diseases (STD) had a higher risk of HIV-1 infection (OR = 1.591, p = 0.024). Recreational drug users had an increased risk of HIV-1 infection (OR = 2.182, p<0.0001).

**Table 3:** Univariate and multivariate logistic regression analysis of risk factors for HIV-1 infection among MSM in Taiwan.

	HIV-1 (+)	HIV-1 (-)	Univariate logistic regression analysis		Multivariate logistic regression analysis	
Variable	N = 201	N = 4474				
	n (%)	n (%)	Odds Ratio	P*	Odds Ratio	P*
**Area**						
**Northern Taiwan**	159 (79.1)	3229 (72.2)	1	1	1	1
**Southern Taiwan**	42 (20.9)	1245 (27.8)	0.685	0.0323	1.507	0.07
**Sexual orientation**						
**Homosexual**	187 (93)	2655 (81.7)	3.775	0.0001	2.617	0.0043
**Bisexual**	9 (4.5)	664 (14.8)	1	1	1	1
**NA**	5 (2.5)	155 (3.5)	2.380	0.1248	2.234	0.1586
**Venue**						
**Gay saunas**	43 (21.4)	1338 (29.9)	2.928	0.0024	2.742	0.0053
**Gay night-clubs**	122 (60.7)	1957 (43.7)	5.679	< .0001	5.116	< .0001
**Party event**	26 (12.9)	268 (6)	8.837	< .0001	6.406	< .0001
**Community centers**	10 (5)	911 (20.4)	1	1	1	1
**Role during anal intercourse**						
**Exclusively insertive**	19 (9.5)	1115 (24.9)	1	1	1	1
**Exclusively receptive**	63 (31.3)	847 (18.9)	4.365	< .0001	3.606	< .0001
**Versatile**	112 (55.7)	2242 (50.1)	2.932	< .0001	2.681	< .0001
**NA**	7 (3.5)	270 (6)	1.521	0.3482	1.708	0.2634
**Number of sexual partners**						
**≤1**	103 (51.2)	2885 (64.5)	1	1	1	1
**>2**	47 (23.4)	520 (11.6)	2.532	< .0001	1.507	0.0399
**NA**	51 (25.4)	1069 (23.9)	1.336	0.0975	1.186	0.3487
**Number of irregular sexual partners**						
**0**	35 (17.4)	1021 (22.8)	1	1		
**≥1**	51 (25.4)	1305 (29.2)	1.140	0.5587		
**NA**	115 (57.2)	2148 (48)	1.561	0.0235		
**Frequency of condom use**						
**Always**	63 (31.3)	1963 (43.9)	1	1	1	1
**Frequently/Occasionally/Rarely/Never**	132 (65.7)	2210 (49.4)	1.861	< .0001	1.509	0.0123
**NA**	6 (3)	301 (6.7)	0.621	0.2702	0.469	0.0842
**Frequency of lubricant use**						
**Always**	123 (61.2)	3135 (70.1)	1	1		
**Frequently/Occasionally/Rarely/Never**	61 (30.4)	1005 (22.5)	1.547	0.0066		
**NA**	17 (8.5)	334 (7.5)	1.297	0.3262		
**Oil-based lubricants during sexual intercourse**						
**Saliva or water-based**	108 (53.7)	2866 (64.1)	1	1	1	1
**Oil-based**	65 (32.3)	973 (21.8)	1.773	0.0004	1.413	0.0409
**NA**	28 (13.9)	635 (14.2)	1.17	0.4681	1.254	0.3465
**Well knowledge of lubricant use**						
**Yes**	71 (35.5)	2006 (44.8)	1	1		
**No**	90 (44.8)	1931 (43.2)	1.317	0.0891		
**NA**	40 (19.9)	537 (12)	2.105	0.0003		
**History of sexually transmitted disease**						
**No**	135 (67.2)	3636 (81.3)	1	1	1	1
**Yes**	32 (15.9)	487 (10.9)	1.770	0.0048	1.591	0.024
**NA**	34 (16.9)	351 (7.9)	2.609	< .0001	1.788	0.0094
**Times of sexual contact per month**						
**< = 1**	41 (20.4)	1532 (34.2)	1	1	1	1
**> = 2**	79 (39.3)	1877 (42)	1.573	0.0206	1.307	0.1746
**NA**	81 (40.3)	1065 (23.8)	2.842	< .0001	2.117	0.0004
**Recreational drug used**						
**No**	77 (38.3)	2638 (59)	1	1	1	1
**Yes**	94 (46.8)	1131 (25.3)	2.848	< .0001	2.182	< .0001
**NA**	30 (14.9)	705 (15.8)	1.458	0.0857	1.27	0.2828

NA: Not available

*, Univariate logistic regression.

We then further investigated the probable factors of the downward trends of HIV-1 prevalence and incidence seen in this study. We used trend analysis to demonstrate the relationship of trend between each factor and time (Table 4). The results showed that from 2013 to 2015, the following four protective factors had a significant increasing trend along with time in MSM populations in Taiwan: correctly use lubricant substitutes, correct knowledge of oil-based lubricant substitutes, always use condoms, and always use condoms after using recreational drugs (p<0.0001; p = 0.02; p<0.0001; p<0.0001, respectively). In contrast, two risk factors including rates of recreational drug use and having more than two sexual partners showed a significant decreasing trend from 2013 to 2015 (p<0.0001; p<0.0001, respectively). Moreover, the results of trend test with incidence were also similar. All of the above-mentioned protective factors had an inverse trend with incidence, which declined year by year. In contrast, the two risk factors showed a decreasing trend similar to that of incidence (Table 4).

**Table 4:** Trend analysis of protect factors and risk factors for HIV-1 infection among MSM in Taiwan during 2013–2015.

	Year				
	2013	2014	2015	P*	
Factors	Yes/No (%)	Yes/No (%)	Yes/No (%)	Time	Incidence
**Incidence (per 100-person year)**	5.97	3.97	2.08		
**Protective factors**					
**Use the right lubricating liquid substitutes**	920/445 (67.4)	945/292 (76.4)	1109/301 (78.7)	<0.0001	<0.0001
**Correct knowledge of lubricating liquid substitutes**	625/672 (48.2)	658/634 (50.9)	794/715 (52.6)	0.02	0.0196
**Always use condom**	626/902 (41)	619/717 (46.3)	781/723 (51.9)	<0.0001	<0.0001
**Always use condom after drug use**^**†**^	82/325 (20.2)	89/220 (28.8)	83/154 (35)	<0.0001	<0.0001
**Risk factors**					
**Use recreational drug**	554/798 (41)	353/896 (28.3)	320/1019 (23.9)	<0.0001	<0.0001
**Have history of STDs**	167 (11.8)	168 (12.6)	184 (12)	0.8879	0.8735
**Play receptive role during anal intercourse**	1167/383 (75.3)	944/360 (72.4)	1153/391 (74.7)	0.6963	0.6757
**Have two or more regular sexual partners**	302/973 (23.7)	140/885 (13.7)	125/1130 (10)	<0.0001	<0.0001

*, Cochran-Armitage trend test with time or incidence, the NA numbers in each variable were be omitted.

^**†**^, Only calculated drug used population.

Analysis of recreational drug use in the Taiwan MSM population showed that 26.2% (1225/4675) of participants enrolled in this study stated that they were recreational drug users (S1 Table). Alkyl nitrites (poppers, RUSH) were the most commonly used drugs (57.1%), followed by 3,4-methylenedioxy-N-methylamphetamine (ecstasy, MDMA) (49.9%), ketamine (45.5%), marijuana (15.5%), amphetamines (12.5%), other drugs (6.7%), lysergic acid diethylamide (LSD) (3.3%), cocaine (1.4%), and heroin (0.7%) (Table 5). More than half of MSM drug users (633/1225, 51.7%) were polydrug users, and only 32.1% (393/1225) were single drug users. The most common drug combinations were ketamine with MDMA (269/633, 42.5%), followed by ketamine with MDMA and sildenafil (Viagra) (73/633, 11.5%), and ketamine with Viagra (52/633, 8.2%).

**Table 5:** Distribution of different types or number of recreational drugs usage among MSM in Taiwan in 2013–2015.

	HIV-1 (+)	HIV-1 (-)	Total	
Variable	N = 94	N = 1131	N = 1225	P^†^
	n (%)	n (%)	n (%)	
**Types**				
**Ketamine**	53 (56.4)	504 (44.6)	557 (45.5)	0.0309
**MDMA**	61 (64.9)	550 (48.6)	611 (49.9)	0.0026
**RUSH**	62 (63.3)	638 (56.3)	700 (57.1)	0.0506
**LSD**	3 (3.1)	38 (3.4)	41 (3.3)	1.0000
**Marijuana**	11 (11.7)	179 (15.8)	190 (15.5)	0.3729
**Amphetamine**	21 (22.3)	132 (11.7)	153 (12.5)	0.0052
**Heroin**	0 (0)	8 (0.7)	8 (0.7)	0.9879
**Cocaine**	4 (4.3)	13 (1.2)	17 (1.4)	0.0356
**Others**	10 (10.6)	72 (6.37)	82 (6.7)	0.1288
**Combinations**				
**Single drug**	9 (9.6)	384 (34)	393 (32.1)	< .0001
**2 Types**				
**Ketamine + MDMA**	25 (26.6)	244 (21.6)	269 (22)	0.2989
**Ketamine + Viagra**	3 (3.2)	49 (4.3)	52 (4.2)	0.7922
**Ketamine + others**	0 (0)	14 (1.2)	14 (1.1)	0.6175
**3 Types**				
**Ketamine + MDMA + Viagra**	12 (12.8)	61 (5.4)	73 (6)	0.0098
**Ketamine + MDMA + others**	1 (1.1)	18 (1.6)	19 (1.6)	1
**Ketamine + Viagra + others**	0 (0)	4 (0.4)	4 (0.3)	1
**More than 4 Types**	2 (2.1)	23 (2)	25 (2)	1
**More than 5 Types**	1 (1)	4 (0.4)	5 (0.4)	0.3296
**Others**	23 (24.5)	149 (13.2)	172 (14)	0.0049
**NA**	18 (19.2)	181 (16)	199 (16.2)	0.466

NA, Not available. MDMA, 3,4-Methylenedioxymethamphetamine. LUSH, Alkyl nitrites. LSD, Lysergic acid diethylamide.

^†^, Fisher exact test.

MSM with exclusively receptive role were associated with higher risk of HIV-1 infection (OR = 2.087, p = 0.0317) (Table 6). Those who did not always use condoms during sexual intercourse were more likely to be infected with HIV-1 than those who always used condoms (OR = 1.823, p = 0.0378). Patrons recruited from gay saunas, gay bars, and party events had significantly higher risk of HIV-1 infection than subjects recruited at community centers (OR = 2.808, p = 0.0315; OR = 6.673, p< 0.0001; OR = 5.343, p = 0.001, respectively). Those who used oil-based lubricants during sex were more likely to be infected with HIV-1 than those who used water-based lubricants (OR = 1.647, p = 0.044). Participants with a history of other STDs were more likely to be infected with HIV-1 (OR = 2.353, p = 0.0023). Unlike the results of univariate logistic regression (S2 Table), amphetamine was the only recreational drug found to be directly associated with increased HIV-1 infection (OR = 1.898, p = 0.0363). In addition, drug combination analysis showed when compared with single drug usage, several polydrug combinations were associated with increased risk of HIV-1 infection, including ketamine with MDMA, ketamine with MDMA and Viagra, and other polydrug combinations (OR = 4.798, p = 0.0004; OR = 5.839, p = 0.0012; OR = 7.279, p<0.0001, respectively).

**Table 6:** Multivariate logistic regression of risk factors for HIV-1 infection among MSM who reported recreational drug usage in Taiwan.

	HIV-1 (+)	HIV-1 (-)		
	N = 94	N = 1131	Odds Ratio	P value*
	n (%)	n (%)		
**Sexual orientation**				
**Homosexual**	89 (94.7)	986 (87.2)	1.979	0.2036
**Bisexual**	3 (3.2)	117 (10.3)	1	1
**NA**	2 (2.1)	28 (2.5)	6.465	0.0418
**Role during anal intercourse**				
**Exclusively insertive**	14 (14.9)	258 (22.8)	1	1
**Exclusively receptive**	33 (35.1)	226 (20)	2.087	0.0317
**Versatile**	47 (50)	624 (55.2)	1.359	0.3291
**NA**	0 (0)	23 (2)	0.083	0.223
**Venue**				
**Gay saunas**	17 (18.1)	272 (24.1)	2.808	0.0315
**Gay night-clubs**	57 (60.6)	468 (41.4)	6.673	< .0001
**Party event**	15 (16)	123 (10.9)	5.343	0.001
**Community centers**	5 (5.3)	268 (23.7)	1	1
**Frequency of condom use**				
**Always**	17 (18.1)	369 (32.6)	1	1
**Frequently/Occasionally/Rarely/Never**	76 (80.9)	718 (63.5)	1.823	0.0378
**NA**	1 (1.1)	44 (3.9)	0.522	0.4442
**Number of sexual partners**				
**≤1**	40 (42.6)	642 (56.8)	1	1
**>2**	34 (36.2)	230 (20.3)	1.479	0.1502
**NA**	20 (21.3)	259 (22.9)	1.259	0.4293
**Oil-based Lubricants during sexual intercourse**				
**Saliva or water-based**	41 (43.6)	693 (61.3)	1	1
**Oil-based**	46 (48.9)	330 (29.2)	1.647	0.044
**NA**	7 (7.5)	108 (9.6)	1.604	0.2739
**History of sexually transmitted disease**				
**No**	61 (64.9)	899 (79.5)	1	1
**Yes**	22 (23.4)	164 (14.5)	2.353	0.0023
**NA**	11 (11.7)	68 (6.0)	1.761	0.1424
**Drug types**				
**Ketamine**				
**No**	41 (43.6)	627 (55.4)	1	1
**Yes**	53 (56.4)	504 (44.6)	0.696	0.1993
**MDMA**				
**No**	33 (35.1)	581 (51.4)	1	1
**Yes**	61 (64.9)	550 (48.6)	1.212	0.5019
**RUSH**				
**No**	31 (33)	494 (43.7)	1	1
**Yes**	63 (67)	637 (56.3)	1.451	0.119
**Amphetamine**				
**No**	73 (77.7)	999 (88.3)	1	1
**Yes**	21 (22.3)	132 (11.7)	1.898	0.0363
**Cocaine**				
**No**	90 (95.7)	1118 (98.9)	1	1
**Yes**	4 (4.3)	13 (1.2)	1.518	0.5352
**Drug combination**				
**Single drug**	9 (9.6)	384 (34)	1	1
**2 Types**				
**Ketamine + MDMA**	25 (26.6)	244 (21.6)	4.798	0.0004
**Ketamine + Viagra**	3 (3.2)	49 (4.3)	2.191	0.247
**Ketamine + others**	0 (0)	14 (1.2)	1.838	0.7054
**3 Types**				
**Ketamine + MDMA + Viagra**	12 (12.8)	61 (5.4)	5.839	0.0012
**Ketamine + MDMA + others**	1 (1.1)	18 (1.6)	6.352	0.0635
**Ketamine + Viagra + others**	0 (0)	4 (0.4)	3.648	0.4647
**4 Types**	2 (2.1)	23 (2)	4.486	0.0693
**More than 4 Types**	1 (1)	4 (0.4)	3.55	0.3292
**Others**	23 (24.5)	149 (13.2)	7.279	< .0001
**NA**	18 (19.2)	181 (16)	4.328	0.0004

NA: Not available. MDMA, 3,4-Methylenedioxymethamphetamine. LUSH, Alkyl nitrites. LSD, Lysergic acid diethylamide.

*, Multivariate logistic regression.

## Discussion

This is the first venue-based surveillance and molecular epidemiology analysis of the MSM populations performed concurrently in southern and northern Taiwan. According to the last annual report from Taiwan Centers for Disease Control (CDC), HIV-1 infected patients from these two regions account for up to 50% of HIV infection cases in Taiwan (northern region including Taipei City: 14.32%, and New Taipei City: 23.05%; southern region indicating Kaohsiung City: 13.82%) [5]. Thus, focusing surveillance and sampling on these regions was essential for obtaining a representative sample for investigation. In addition to the investigation of possible risk factors of HIV-1 infection through demographic and behavioral data, a phylogenetic tree was used to investigate the interactions between the social networks of the MSM populations in southern and northern Taiwan.

From 2013 to 2015, the HIV-1 incidence rate and prevalence rate of the MSM population in Taiwan were on a decreasing trend. This may be associated with the changes in infection-related behavioral risk factors and protective factors of the population in different years as seen in our study. The results of trend test showed that protective factors such as correctly using water-based lubricants and always using condoms during sexual intercourse significantly increased each year, whereas risk factors such as using recreational drugs and having two or more regular sexual partners significantly decreased each year (Table 4). Furthermore, we also found that the increasing trend of protective factors and the decreasing trend of risk factors among the MSM population were significantly associated with the decreasing trend of incidence. These trends may be attributed to the efforts of governmental and non-governmental organizations such as local LGBTQ centers and AIDS prevention organizations on health education and promotion. Interestingly, ever since Taiwan CDC started to promote patient education such as “incorrect use of oil-based lubricants increases the risk of HIV-1 infection” in 2014, subjects have had increased knowledge for differentiating between water-based and oil-based lubricant substitutes. However, based on the results of multivariate logistic regression analysis, this knowledge did not directly affect the risk of HIV-1 infection. 12.4% of subjects still used oil-based lubricants during sex even though they had correct knowledge (257/2077). This gap between knowledge and practice “to know is easier than to do” needs to be kept in mind. As we know, people using oil-based lubricants have an increased probability of condom rupture during sexual intercourse. Moreover, 14.3% of subjects always using condoms stated that they used oil-based lubricants during sex (289/2026). This may offset the protective effect of the “good” behavior that subjects take. It is therefore imperative for the relevant health authorities to implement intervention and promotion programs.

The phylogenetic tree analysis showed that the interaction between the MSM populations in northern and southern Taiwan could cause cross-regional transmission of the HIV-1. At the same time, we also discovered that local HIV-1 strains had significant *env* sequence correlations with Thailand and Hong Kong strains, suggesting that HIV-1 may be transmitted through factors such as tourism and business events, and indirectly transmitted to the Taiwanese MSM population. In addition, from past observations, the dominantHIV-1 subtype of the MSM population in Taiwan is subtype B [21, 22]. The dominant HIV-1 subtype of sex workers, prostitute customers, and heterosexual populations is CRF01_AE. HIV-1 subtype analysis of this study revealed subjects with double infections of subtype B and CRF 01_AE, which represents the possibility of transmission across different risk populations. The transmission across these regions or risk populations may indirectly increase the diversity of the local HIV-1 gene bank, which may in turn increase the possibility of HIV-1 genetic mutations or recombination. This needs close monitoring and the relevant authorities need to be notified with firsthand information in order to implement proper preventive measures.

In depth investigation of the recreational drug users in the MSM population, our results are in agreement with previous reports that polydrug users were more prone to engage in unprotected sexual behavior and were therefore at a higher risk of HIV-1 infection [23, 24]. Comparison of the sexual behaviors between the general MSM population and the MSM population using recreational drugs (Tables 3 and 6) showed that the percentages of subjects not always using condoms during sexual intercourse (2342/4675, 50.1% and 794/1225, 64.8%, respectively) and those using oil-based lubrincants (1038/4675, 22.2% and 376/1225, 30.7%, respectively) were quite different. In addition, we found that in addition to the one known combination of ketamine with MDMA and Viagra, which is known to increase the risk of HIV-1 infection, the combination of ketamine with MDMA could also increase the risk of infection. We also found that other unknown combination sets of polydrug use could increase the risk of HIV-1 infection and merit further investigation. Multivariate regression analysis revealed similar results to analysis in the general MSM population (Tables 3 and 6), except that sexual orientation and sexual roles did not affect the risk of HIV-1 infection in this subpopulation using recreational drugs. If not considering the combination patterns but focusing on the drug types only, we found that the use of amphetamines also significantly increased the risk of HIV-1 infection. Patterson et al. also reported that amphetamine users were mostly polydrug users, and these heavy polydrug users were involved in more high-risk behaviors [25]. This was consistent with our observation that 64.3% (99/154) of amphetamine users were polydrug users, while 49.9% (534/1071) of other drug users were polydrug users. In brief, the use of multiple drugs greatly influences the risk of HIV-1 infection, and is worthy of in depth monitoring and intervention.

Our study results showed a gradual decline in prevalence and incidence rates of HIV-1 infection from 2013 to 2015. Ideally, if we can sample from a known sample frame of a critical population such as MSM, the epidemiology index of HIV-1 infection including prevalence, incidence and behavioral risk factors could be determined precisely, and could be further used in prevention strategy planning, cost-effectiveness calculations, and target intervention designs [16, 26]. However, MSM are actually composed of subgroups of individuals with very different experiences, social environments and behaviors [27, 28]. Therefore, the most appropriate sampling method to reach the entirety sample frame is not easy to define, and that is also the major limitation of our study. We recruited participants from gay bars, party events, gay saunas and community centers, which theoretically cover the major social circles of MSM; however, there is the possibility sampling bias in these high-risk venues [29]. With the advent of online social platforms including community websites and mobile applications for sex-seeking in recent years [9, 30], it is possible that the next generation of MSM no longer frequent attends the traditional venues and we may have missed a fraction of potential subjects using these new social platforms. Furthermore, research has shown that the increased use of mobile-based social network applications for sex seeking online was correlated with unprotected sex [31, 32].

In conclusion, the HIV-1 prevalence and incidence of the MSM population seeking sex at high-risk social venues decreased from 2013 to 2015. This was associated with the trends of several protective or risk behaviors such as correct use of water-based lubricants and recreational drug use. The evidences in phylogenetic analysis revealed cross-regional and international transmission. Online social networking, especially mobile-based applications, may have become an overlooked area of surveillance, education and disease prevention. Future studies need to consider these factors when surveying MSM.

## Supporting information

S1 Table Demographic data of patrons who reported recreational drug use from different gay venues participated in this study. (DOCX)

S2 Table Univariate analysis of risk factors for HIV-1 infection among MSM who reported recreational drug use in Taiwan. (DOCX)
