# Supplementary material for: Molecular epidemiology of HIV-1 infection among men who have sex with men in Taiwan from 2013 to 2015
Source: PLoS One. 2018 Dec 6;13(12):e0202622. doi: 10.1371/journal.pone.0202622 (PMC6283607; doi:10.1371/journal.pone.0202622)
Supplement: S1 Table — (DOCX) [file pone.0202622.s001.docx]

**S1 Table. Demographic data of patrons who reported recreational drug use from different gay venues participated in this study.**

|  | **HIV-1 (+)** | **HIV-1 (-)** |  |
| --- | --- | --- | --- |
| **Variable** | **N = 94** | **N = 1131** | **P** |
|  | **n (%)** | **n (%)** |  |
| **Years** |  |  | 0.0005^†^ |
| **2013** | 59 (62.8) | 493 (43.6) |  |
| **2014** | 23 (24.5) | 330 (29.2) |  |
| **2015** | 12 (12.8) | 308 (27.2) |  |
| **Area** |  |  | 0.6418^†^ |
| **North of Taiwan** | 83 (88.3) | 975 (86.2) |  |
| **South of Taiwan** | 11 (11.7) | 156 (13.8) |  |
| **Age** |  |  | 0.7036 |
| **18-29** | 46 (48.9) | 541 (47.8) |  |
| **30-39** | 34 (36.2) | 404 (35.7) |  |
| **40-49** | 7 (7.5) | 105 (9.3) |  |
| **≧50** | 0 (0) | 20 (1.8) |  |
| **NA** | 7 (7.5) | 61 (5.4) |  |
| **Occupation** |  |  | 0.0488^*^ |
| **Student** | 18 (19.2) | 174 (15.4) |  |
| **Government employees** | 10 (10.6) | 98 (8.7) |  |
| **Office worker** | 56 (59.6) | 658 (58.2) |  |
| **Specialist** | 3 (3.2) | 82 (7.3) |  |
| **Unemployed/Other** | 4 (4.3) | 110 (9.7) |  |
| **NA** | 3 (3.2) | 9 (0.8) |  |
| **Education** |  |  | 0.2844^†^ |
| **≤Junior high school** | 2 (2.1) | 15 (1.3) |  |
| **Senior high school** | 18 (19.2) | 173 (15.3) |  |
| **College** | 65 (69.2) | 780 (69) |  |
| **≧Graduate** | 8 (8.5) | 157 (13.9) |  |
| **NA** | 1 (1.1) | 6 (0.5) |  |
| **Marital status** |  |  | 0.0006^†^ |
| **Single** | 81 (86.2) | 1082 (95.7) |  |
| **Married** | 4 (4.3) | 12 (1.1) |  |
| **Divorced/Separated/Widowed** | 1 (1.1) | 13 (1.2) |  |
| **NA** | 8 (8.5) | 24 (2.1) |  |
| **Sexual orientation** |  |  | 0.0541^†^ |
| **Homosexual** | 89 (94.7) | 986 (87.2) |  |
| **Bisexual** | 3 (3.2) | 117 (10.3) |  |
| **NA** | 2 (2.1) | 28 (2.5) |  |
| **Venue** |  |  | <.0001^†^ |
| **Gay saunas** | 17 (18.1) | 272 (24.1) |  |
| **Gay night-clubs** | 57 (60.6) | 468 (41.4) |  |
| **Party event** | 15 (16) | 123 (10.9) |  |
| **Community centers** | 5 (5.3) | 268 (23.7) |  |

NA, Not available.

^*^, Chi-square test.

^†^, Fisher exact test.
