# Supplementary material for: Molecular epidemiology of HIV-1 infection among men who have sex with men in Taiwan from 2013 to 2015
Source: PLoS One. 2018 Dec 6;13(12):e0202622. doi: 10.1371/journal.pone.0202622 (PMC6283607; doi:10.1371/journal.pone.0202622)
Supplement: S2 Table — (DOCX) [file pone.0202622.s002.docx]

**S2 Table. Univariate analysis of risk factors for HIV-1 infection among MSM who reported recreational drug use in Taiwan.**

|  | **HIV-1 (+)** | **HIV-1 (-)** |  |  |
| --- | --- | --- | --- | --- |
| **Variable** | **N = 94** | **N = 1131** | **Odds Ratio** | **P^*^** |
|  | **n (%)** | **n (%)** |  |  |
| **Area** |  |  |  |  |
| **Northern Taiwan** | 83 (88.3) | 975 (86.2) | 1 | 1 |
| **Southern Taiwan** | 11 (11.7) | 156 (13.8) | 0.828 | 0.5709 |
| **Sexual orientation** |  |  |  |  |
| **Homosexual** | 89 (94.7) | 986 (87.2) | 3.520 | 0.0344 |
| **Bisexual** | 3 (3.2) | 117 (10.3) | 1 | 1 |
| **NA** | 2 (2.1) | 28 (2.5) | 2.786 | 0.2741 |
| **Venue** |  |  |  |  |
| **Gay saunas** | 17 (18.1) | 272 (24.1) | 3.350 | 0.0191 |
| **Gay night-clubs** | 57 (60.6) | 468 (41.4) | 6.528 | <.0001 |
| **Party event** | 15 (16) | 123 (10.9) | 6.536 | 0.0004 |
| **Community centers** | 5 (5.3) | 268 (23.7) | 1 | 1 |
| **Role during anal intercourse** |  |  |  |  |
| **Exclusively insertive** | 14 (14.9) | 258 (22.8) | 1 | 1 |
| **Exclusively receptive** | 33 (35.1) | 226 (20) | 2.691 | 0.0028 |
| **Versatile** | 47 (50) | 624 (55.2) | 1.388 | 0.2954 |
| **NA** | 0 (0) | 23 (2) | <0.001 | 0.9871 |
| **Number of sexual partners** |  |  |  |  |
| **≤1** | 40 (42.6) | 642 (56.8) | 1 | 1 |
| **>2** | 34 (36.2) | 230 (20.3) | 2.373 | 0.0004 |
| **NA** | 20 (21.3) | 259 (22.9) | 1.239 | 0.4492 |
| **Number of irregular sexual partners** |  |  |  |  |
| **0** | 12 (12.8) | 194 (17.2) | 1 | 1 |
| **≥1** | 36 (38.3) | 488 (43.2) | 1.193 | 0.6086 |
| **NA** | 46 (48.9) | 449 (39.7) | 1.656 | 0.1324 |
| **Frequency of condom use** |  |  |  |  |
| **Always** | 17 (18.1) | 369 (32.6) | 1 | 1 |
| **Frequently/Occasionally/**  **Rarely/Never** | 76 (80.9) | 718 (63.5) | 2.298 | 0.0026 |
| **NA** | 1 (1.1) | 44 (3.9) | 0.493 | 0.4974 |
| **Frequency of lubricant use** |  |  |  |  |
| **Always** | 45 (47.9) | 702 (62.1) | 1 | 1 |
| **Frequently/Occasionally/**  **Rarely/Never** | 44 (46.8) | 388 (34.3) | 1.769 | 0.0099 |
| **NA** | 5 (5.3) | 41 (3.6) | 1.902 | 0.1966 |
| **Oil-based lubricants during sexual intercourse** |  |  |  |  |
| **Saliva or water-based** | 41 (43.6) | 693 (61.3) | 1 | 1 |
| **Oil-based** | 46 (48.9) | 330 (29.2) | 2.356 | 0.0001 |
| **NA** | 7 (7.5) | 108 (9.6) | 1.096 | 0.8288 |
| **Well knowledge of lubricant use** |  |  |  |  |
| **Yes** | 31 (33) | 455 (40.2) | 1 | 1 |
| **No** | 46 (48.9) | 562 (49.7) | 1.201 | 0.4461 |
| **NA** | 17 (18.1) | 114 (10.1) | 2.189 | 0.0142 |
| **History of sexually transmitted disease** |  |  |  |  |
| **No** | 61 (64.9) | 899 (79.5) | 1 | 1 |
| **Yes** | 22 (23.4) | 164 (14.5) | 1.977 | 0.0095 |
| **NA** | 11 (11.7) | 68 (6.0) | 2.384 | 0.0133 |
| **Times of sexual contact per month** |  |  |  |  |
| **<=1** | 16 (17) | 318 (28.1) | 1 | 1 |
| **>=2** | 43 (45.7) | 571 (50.5) | 1.497 | 0.1804 |
| **NA** | 35 (37.2) | 242 (21.4) | 2.875 | 0.0008 |
| **Drug types** |  |  |  |  |
| **Ketamine** |  |  |  |  |
| **No** | 41 (43.6) | 627 (55.4) | 1 | 1 |
| **Yes** | 53 (56.4) | 504 (44.6) | 1.608 | 0.0281 |
| **MDMA** |  |  |  |  |
| **No** | 33 (35.1) | 581 (51.4) | 1 | 1 |
| **Yes** | 61 (64.9) | 550 (48.6) | 1.953 | 0.0028 |
| **RUSH** |  |  |  |  |
| **No** | 31 (33) | 494 (43.7) | 1 | 1 |
| **Yes** | 63 (67) | 637 (56.3) | 1.576 | 0.0455 |
| **LSD** |  |  |  |  |
| **No** | 91 (96.8) | 1093 (96.6) | 1 | 1 |
| **Yes** | 3 (3.2) | 38 (3.4) | 0.948 | 0.9305 |
| **Marijuana** |  |  |  |  |
| **No** | 83 (88.3) | 952 (84.2) | 1 | 1 |
| **Yes** | 11 (11.7) | 179 (15.8) | 0.705 | 0.2907 |
| **Amphetamine** |  |  |  |  |
| **No** | 73 (77.7) | 999 (88.3) | 1 | 1 |
| **Yes** | 21 (22.3) | 132 (11.7) | 2.177 | 0.0032 |
| **Heroin** |  |  |  |  |
| **No** | 94 (100) | 1123 (99.3) |  |  |
| **Yes** | 0 (0) | 8 (0.7) | <0.001 | 0.9879 |
| **Cocaine** |  |  |  |  |
| **No** | 90 (95.7) | 1118 (98.9) | 1 | 1 |
| **Yes** | 4 (4.3) | 13 (1.2) | 3.822 | 0.0213 |
| **Others** |  |  |  |  |
| **No** | 84 (89.4) | 1059 (93.6) | 1 | 1 |
| **Yes** | 10 (10.6) | 72 (6.4) | 1.751 | 0.1156 |
| **Drug combination** |  |  |  |  |
| **Single drug** | 9 (9.6) | 384 (34) | 1 | 1 |
| **2 Types** |  |  |  |  |
| **Ketamine + MDMA** | 25 (26.6) | 244 (21.6) | 4.372 | 0.0002 |
| **Ketamine + Viagra** | 3 (3.2) | 49 (4.3) | 2.612 | 0.1602 |
| **Ketamine + others** | 0 (0) | 14 (1.2) | <0.001 | 0.9856 |
| **3 Types** |  |  |  |  |
| **Ketamine + MDMA + Viagra** | 12 (12.8) | 61 (5.4) | 8.393 | <.0001 |
| **Ketamine + MDMA + others** | 1 (1.1) | 18 (1.6) | 2.37 | 0.4248 |
| **Ketamine + Viagra + others** | 0 (0) | 4 (0.4) | <0.001 | 0.9923 |
| **More than 4 Types** | 2 (2.1) | 23 (2) | 3.71 | 0.1058 |
| **More than 5 Types** | 1 (1) | 4 (0.4) | 10.667 | 0.0427 |
| **Others** | 23 (24.5) | 149 (13.2) | 6.586 | <.0001 |
| **NA** | 18 (19.2) | 181 (16) | 4.243 | 0.0005 |

NA: Not available. MDMA, 3,4-Methylenedioxymethamphetamine. LUSH, Alkyl nitrites. LSD, Lysergic acid diethylamide.

^*^, Univariate logistic regression.
